# Into the Deep: New Data on the Lipid and Fatty Acid Profile of Redfish *Sebastes mentella* Inhabiting Different Depths in the Irminger Sea

**DOI:** 10.3390/biom11050704

**Published:** 2021-05-09

**Authors:** Viktor P. Voronin, Nina N. Nemova, Tatjana R. Ruokolainen, Dmitrii V. Artemenkov, Aleksei Y. Rolskii, Alexei M. Orlov, Svetlana A. Murzina

**Affiliations:** 1 Institute of Biology of the Karelian Research Centre of the Russian Academy of Sciences (IB KarRC RAS), 11 Pushkinskaya Street, 185910 Petrozavodsk, Russia; nemova@krc.karelia.ru (N.N.N.); truok@krc.karelia.ru (T.R.R.); 2 Russian Federal Research Institute of Fisheries and Oceanography (VNIRO), 17 V. Krasnoselskaya Street, 107140 Moscow, Russia; artemenkov@vniro.ru; 3 Polar Branch of the “Russian Federal Research Institute of Fisheries and Oceanography (VNIRO)” (“PINRO” named after N.M. Knipovich), 6 Akademika Knipovicha Street, 183038 Murmansk, Russia; rol-lex@mail.ru; 4 Shirshov Institute of Oceanology of the Russian Academy of Sciences (IO RAS), 36 Nakhimovsky Prospekt, 117997 Moscow, Russia; orlov@vniro.ru; 5 A.N. Severtsov Institute of Ecology and Evolution of the Russian Academy of Sciences (IPEE RAS), 33 Leninsky Prospekt, 119071 Moscow, Russia; 6 Tomsk State University (TSU), 36 Lenin Avenue, 634050 Tomsk, Russia

**Keywords:** lipids, fatty acids, beaked redfish, *Sebastes mentella*, mesopelagic zone, Arctic, North Atlantic

## Abstract

New data on lipid and fatty acid profiles are presented, and the dynamics of the studied components in muscles in the males and females of the beaked redfish, *Sebastes mentella*, in the depth gradient of the Irminger Sea (North Atlantic) is discussed. The contents of the total lipids (TLs), total phospholipids (PLs), monoacylglycerols (MAGs), diacylglycerols (DAGs), triacylglycerols (TAGs), cholesterol (Chol), Chol esters, non-esterified fatty acids (NEFAs), and wax esters were determined by HPTLC; the phosphatidylserine (PS), phosphatidylethanolamine (PE), phosphatidylinositol (PI), phosphatidylcholine (PC), and lysophosphatidylcholine (LPC) were determined by HPLC; and fatty acids of total lipids were determined using GC. The Chol esters prevailed in muscles over the storage TAGs, and the wax ester content was high, which is a characteristic trait of vertically migrating species. Specific dynamics in certain PL in redfish were found to be depended on depth, suggesting that PLs are involved in the re-arrangement of the membrane physicochemical state and the maintenance of motor activity under high hydrostatic pressure. The high contents of DHA and EPA were observed in beaked redfish muscles is the species’ characteristic trait. The MUFAs in muscles include dietary markers of zooplankton (copepods)—20:1(n-9) and 22:1(n-11), whose content was found to be lower in fish sampled from greater depths.

## 1. Introduction

Lipids are essential multifunctional components of organisms. Lipids and their fatty acids are regarded as quite labile biochemical molecules that participate in many complex compensatory reactions by which an organism maintains a homeostasis of interrelated metabolic processes in response to certain environmental factors or a combination thereof. Individual lipid classes can perform several functions related to adaptation processes in specific eco-physiological situations [1,2,3,4,5,6,7,8,9,10,11].

Deepwater organisms, inhabiting depths from 200 to 1000 m, are exposed to a range of environmental factors that often form extremely challenging combinations, e.g., high hydrostatic pressures low temperatures, and special photoperiods. The successful adaptation of marine organisms, in particular fish, to living in such environments is facilitated by a variety of biochemical mechanisms, including those involving lipids. Research has demonstrated [1,12,13], for instance, that adaptations in the brain membrane lipids in fish living under high hydrostatic pressure are similar to the adaptations induced by temperature decline. Relevant buoyancy deep down a water column is known to be maintained due to a high content and variations of neutral lipids (cholesterol esters, triacylglycerols, and wax esters), which is typical in vertically migrating species [14,15]. Some researchers [4,6,16] have found that, compared to warm-water fish, cold-water fish had a higher content of omega-3 polyunsaturated fatty acids than omega-6 and omega-9 fats, and this has been attributed to the organism’s adaptive response to water temperature changes through the modification of the cell membrane microviscosity. Many researchers [1,17,18] associate the elevated content of docosahexaenoic acid (a physiologically significant omega-3 fatty acid) with a higher locomotor activity. Another supposition is that 18:1 fatty acids have active roles in the compensatory response to changes in the temperature and depth factors [6,19,20]. In addition, lipid and fatty acid qualitative and quantitative spectra, as well as their individual components (including marker fatty acids), can also serve as organ-specific and species-specific indicators [21,22,23,24,25,26]. Recent papers have illustrated a wide application of the association between the content of lipids and their profiles (separately or together with genetic markers) in studies of species’ population structures, both in fish and in humans [27,28,29,30,31,32,33,34].

One of the most common and commercially valuable redfish species in the North Atlantic region is the beaked redfish, *Sebastes mentella* [35,36,37,38,39]. Its range extends from the Barents and Greenland Seas to the Canadian coast via central parts of the North Atlantic [40,41,42,43], and the largest population is found in the Irminger Sea [38,44,45,46]. The depth range inhabited by the beaked redfish extends down to more than 1000 m [42,47,48]. The species’ biology, ecology, population structure, and distribution have been addressed in a number of papers [23,39,42,46,47,48,49,50,51,52]; however, some aspects remain debatable [23,39,46,49,50,51]. Far less studied are the redfish’s ecological biochemical adaptations down the depth gradient. Though some studies on changes in the lipid metabolism of beaked redfish under extreme yet stable environmental conditions are available [21,22,23,24,53], they have not supplied full knowledge of the lipid and fatty acid spectrum in redfish at different depths. The presented paper offers a comparative analysis of the lipids and their fatty acid components in beaked redfish males and females inhabiting a depth gradient of 250–700 m in the Irminger Sea (North Atlantic), providing new information about the biochemical mechanisms of adaptations to mesopelagic life in northern-sea fish.

## 2. Materials and Methods

### 2.1. Sample Collection

**Figure 1:**
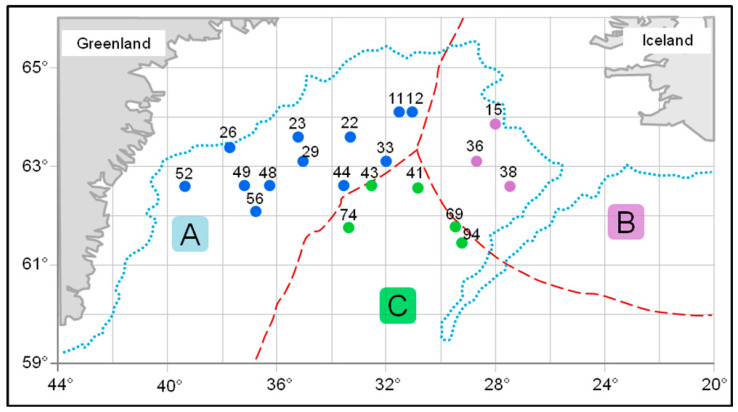
Schematic map of the collection of the beaked redfish, *Sebastes mentella,* in the Irminger Sea (North Atlantic). Legend: A—Greenland fishing zone; B—Iceland’s exclusive economic zone; C—NEAFC regulation area; points—trawl numbers.

Muscle tissue samples from male and female beaked redfish, *Sebastes mentella*, were obtained during research in the Irminger Sea (59°60′–64°60′ N and 26°20′–41°50′ W) in summer (June–July) aboard the R/V Atlantida (Figure 1 and Table 1). The species identification was conducted according to a recommended guide [54]. Biomaterials were collected in three areas (NEAFC regulation area, Greenland fishing zone, and Iceland’s exclusive economic zone) at depths of 250, 325, 375, 400 (only females—2), 650, and 700 m.

The samples were collected according to the Manual for the Implementation of the International Deep Sea Pelagic Ecosystem Survey in the Irminger Sea and Adjacent Waters, developed and approved by the ICES Working Group on International Deep Pelagic Ecosystem Surveys (WGIDEEPS) [55]. To carry out trawling operations on the R/V Atlantida, a mid-water trawl of 78.7/416 m with project 2492-02 was used, the rope and net parts of which were made of modern lightweight materials; the mesh size in the wings was 68 mm, and the cod end was 16 mm.

The collected samples were studied under the cooperation agreement between the Federal Agency for Fisheries and the Federal State Budgetary Institution of the Russian Academy of Sciences, based on the Joint Scientific Research Program of the Federal Agency for Fishery and the Russian Academy of Sciences.

### 2.2. Lipid Extraction and Analysis

The total lipids (TLs) from muscle tissue were extracted using the Folch method chloroform–methanol (2:1, *v/v*) solvent system [56].

#### 2.2.1. Neutral Lipids Analysis

The qualitative and quantitative determination of individual lipid classes—total phospholipids (PLs), monoacylglycerols (MAGs), diacylglycerols (DAGs), triacylglycerols (TAGs), cholesterol esters (Chol esters), cholesterol (Chol), non-esterified fatty acids (NEFAs), and wax esters—was carried out using high-performance thin-layer chromatography (HPTLC). The fractionation of total lipids was carried out on ultrapure glass-based plates—HPTLC Silicagel 60 F_254_ Premium Purity (Merck, Germany). The application of microvolumes of the sample was performed using a semi-automatic Linomat 5 applicator (CAMAG, Switzerland), and the separation of individual lipid classes was carried out using an ADC2 chromatographic chamber (CAMAG, Switzerland) in the solvent system of hexane-diethyl ether-acetic acid (32:8:0.8, *v*/*v*), with a supersaturated zinc nitrate (ZnNO_3_*6H_2_O) solution used for maintaining humidity (47–49% humidity) [57]. The formation of visible individual lipid spots was observed in a solution of copper sulfate (CuSO_4_) with orthophosphoric acid (H_3_PO_4_), followed by the heating of the plate to 160 °C for 15 min. The qualitative and quantitative determination of lipid components was carried out in the chamber of a TLC Scanner 4 densitometer (CAMAG, Switzerland) [58]. The identification of individual lipid classes was carried out according to the standards of the respective studied components (Sigma-Aldrich USA) while taking the correspondence of the Rf values into account. 

#### 2.2.2. Polar Lipids Analysis

The qualitative and quantitative determination of individual phospholipid fractions—phosphatidylcholine (PC), phosphatidylethanolamine (PEA), phosphatidylserine (PS), phosphatidylinositol (PI), and lysophosphatidylcholine (LPC)—was performed by high-performance liquid chromatography (HPLC) and previously described in [11]. Phospholipid standards (Sigma-Aldrich, USA) were used for identifying and quantifying the phospholipid compounds in the sample. We identified six phospholipids (as total certain fractions without focusing on molecular species): PS, PE, PI, PC, LPC, and SM.

#### 2.2.3. Fatty Acids Analysis

The qualitative and quantitative fatty acid (FA) profiles of the TLs were analyzed by gas–liquid chromatography (GC). Fatty acid methyl esters (FAMEs) were separated on a Chromatek-Crystall-5000.2 gas chromatograph with a flame-ionization detector (FID) and an automatic liquid dispenser (Chromatek, Yoshkar-Ola, Russia). The separation of FAs was carried out for 120 min in an isothermal configuration (200 °C) on a Zebron ZB-FFA capillary column (Phenomenex, USA) using nitrogen as a mobile phase. The Chromatek-Analytik-5000.2 software Chromatek Analytic V. 3.0.298.1 (Chromatek, Yoshkar-Ola, Russia) was used according to the analytic procedure described in [11]. FAMEs were identified with the standard mixtures of the Supelco 37 Component FAME mix, bacterial acid methyl ester (BAME), and polyunsaturated fatty acid (PUFA) No. 1 (Sigma-Aldrich, USA).

### 2.3. Statistical Analysis

To perform statistical analysis, the free R-programming language (v. 3.6.1.) with basic packages and additional readxl (v. 1.3.1), tidyverse (v. 1.3.0), corrgram (v. 1.13), factoextra (v.1.0.6), randomForest (v. 4.6-14), quantreg (v. 5.52), cowplot (v. 1.1.1), and gmodels (v. 2.18.1) packages was used. Statistically significant differences between depths were tested by the non-parametric Kruskal–Wallis test, and between individual depth differences were tested by the Wilcoxon Mann–Whitney test [59]. Statistical significance was set at *p* ≤ 0.05. Machine learning was carried out using the random forest classification; the mean decrease Gini coefficient was used to determine the significant classifiers [60]. Multivariate analysis was performed using principal components analysis for FAs, the content of which was more than 1% of the total FA [24].

The biochemical analysis was performed at the Scientific Center collective usage platform of the Karelian Research Centre of the Russian Academy of Sciences.

## 3. Results

### 3.1. Species-Specific Characteristics of the Total Lipids (TLs) in Beaked Redfish, Their Sex Specificity, and Change Depth-Wise

**Figure 2:**
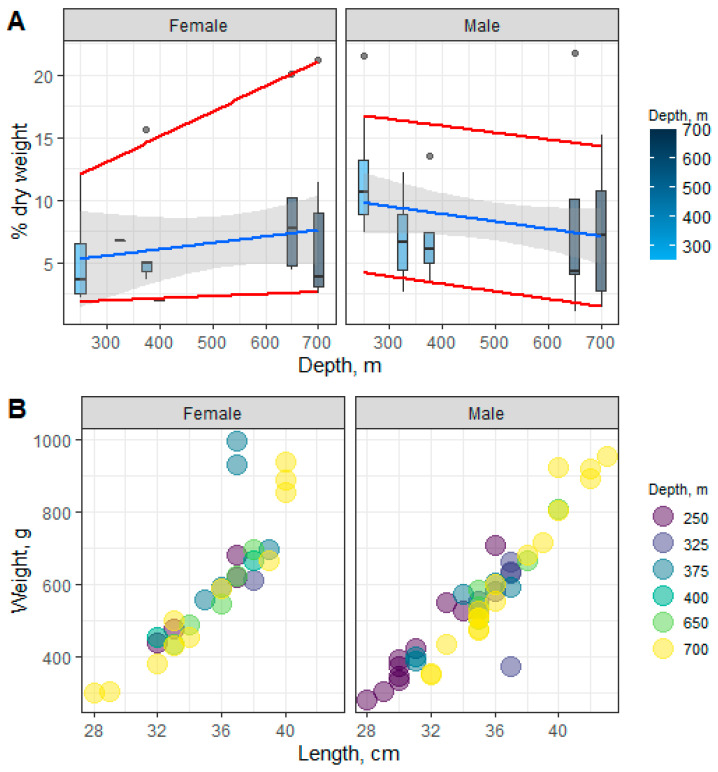
Changes in the content of the total lipids (**A**) and size–weight characteristics (**B**) in male and female beaked redfish, *Sebastes mentella,* in the depth gradient of the Irminger Sea (North Atlantic).

The total lipid content ranged from 7.09 to 11.87% dry weight in males and from 2.01 to 9.47% dry weight in females at 250, 325, 375, 400, 650, and 700 m of depth. Changes in the muscle TL content in males and females across the sampled depths are shown in Figure 2A. Significant differences in the TL content in muscles in males and females along the depth gradient were not detected. That said, depth-wise trend lines (linear regressions—the median and quantiles for the 10th and 90th percentiles) followed the opposite directions in males and females due to differences in individual lipid classes: monoacylglycerols (MAGs), cholesterol (Chol), non-esterified fatty acid (NEFA), wax esters, and cholesterol esters (Chol esters) at 250 m of depth—0.33 ± 0.05 and 0.15 ± 0.06, 1.45 ± 0.15 and 0.66 ± 0.30, 0.35 ± 0.04 and 0.17 ± 0.06, 1.27 ± 0.17 and 0.52 ± 0.27, and 4.86 ± 0.67 and 2.01 ± 0.79% dry weight for males and females, respectively. The observed changes correlate with the length–weight characteristics of beaked redfish and their depth-wise distribution (Figure 2B).

### 3.2. Species-Specific Characteristics of Neutral Lipids in Beaked Redfish, Their Sex Specificity and Change Depth-Wise

**Figure 3:**
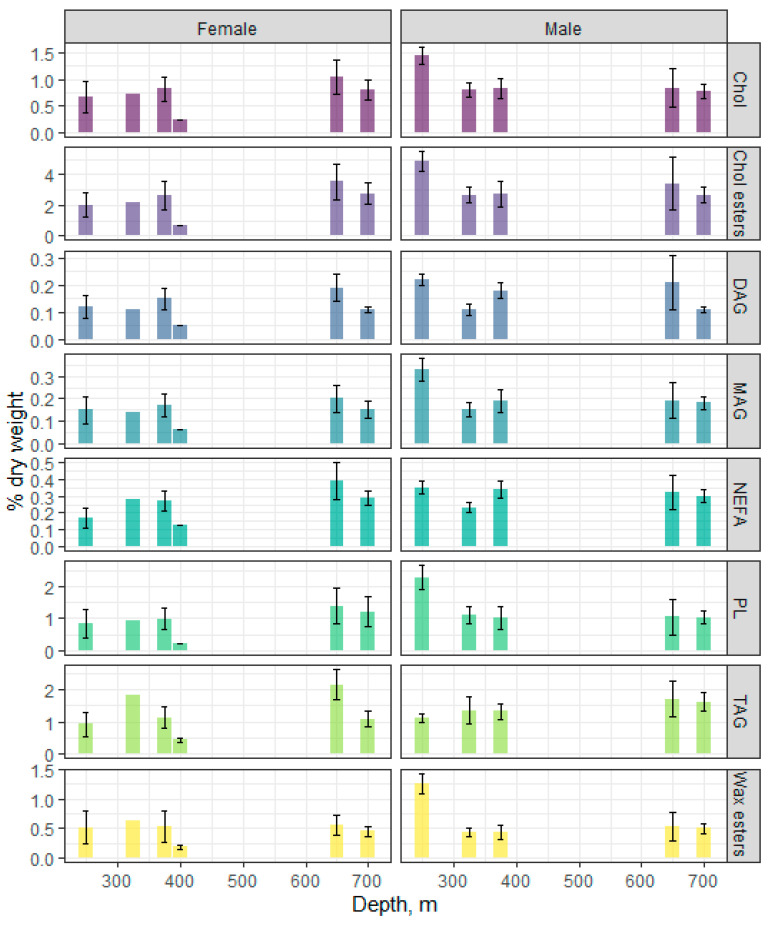
Changes in the content of individual lipid classes in the males and females of beaked redfish, *Sebastes mentella,* in the depth gradient of the Irminger Sea (North Atlantic).

Figure 3 shows the profiles for individual lipid classes and their dynamics in the male and female muscles at different depths. A dominance of Chol esters was detected—from 2.68 to 4.86 and from 0.67 to 3.57% dry weight in males and females, respectively, with significant differences between sexes observed only at the depth of 250 m. There was no significant change in the Chol ester content in muscles with depth, except for a significant increase in males at 250 m versus other depths.

The content of the triacylglycerols (TAGs) and total phospholipids (PLs) in muscles varied within 1.12–1.70 and 1.01–2.27% dry weight and within 0.44–2.14 and 0.23–1.39% dry weight in males and in females, respectively. Only males from the 250 m depth showed a prevalence of PLs over TAGs (2.27 vs 1.12% dry weight, respectively). The content of wax esters in the muscle tissue of males and females was high (up to 10% of the sum of all lipid classes) and did not significantly change with depth—from 0.44 to 1.27 and from 0.19 to 0.56% dry weight for males and females, respectively.

The application of the machine learning (random forest algorithm) to lipid classes (total PLs, MAGs, diacylglycerols (DAGs), TAGs, Chol, Chol esters, NEFAs, and wax esters) revealed that the strongest classifiers (according to mean decrease Gini—MDG) for both sexes in this fish species over the depth gradient were DAGs (MDG = 7.23), NEFAs (MDG = 6.34), Chol (MDG = 5.67), and wax esters (MDG = 5.41), thus indicating that changes in these lipids are associated with the depth at which the fish lives. An estimation of the differences between groups of fish by the Kruskal–Wallis non-parametric test only revealed significant differences for DAGs and wax esters in males.

### 3.3. Species-Specific Characteristics of Polar Lipids in Beaked Redfish, Their Sex Specificity, and Change in the Depth Gradient

**Figure 4:**
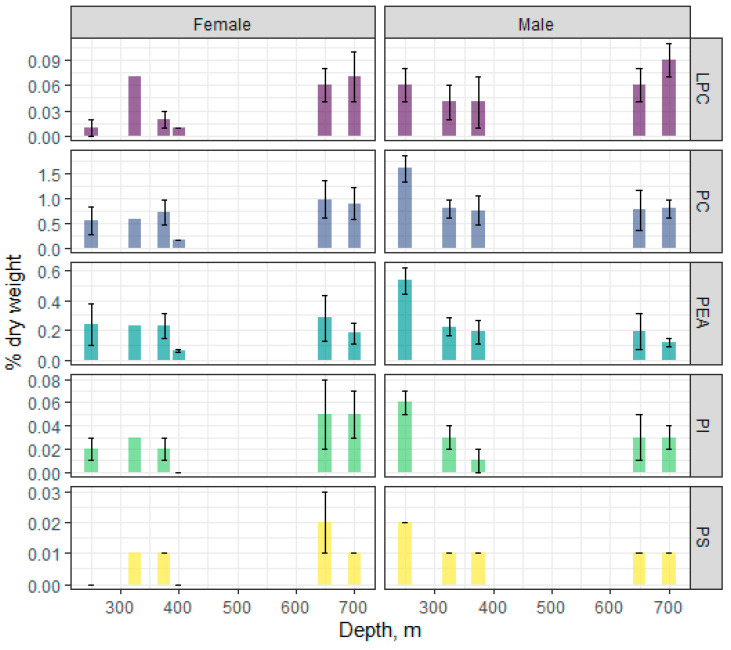
Changes in the content of individual phospholipid classes in males and females of the beaked redfish, *Sebastes mentella,* in the depth gradient of the Irminger Sea (North Atlantic).

Among fractions of polar lipids (total phospholipids), phosphatidylcholine (PC) and phosphatidylethanolamine (PEA) (Figure 4) dominated, the content of which varied within 0.76–1.6 and 0.12–0.53% dry weight, respectively, in males and 0.16–0.98 and 0.06–0.28% dry weight, respectively, in females. An analysis showed that PC and PEA in the fish correlated at r = 0.98 with each other and inversely correlated with depth (r = −0.96 and −0.98, respectively), suggesting a concerted decline in these major membrane lipids with depth. No significant changes of the PC and PEA contents with depth were observed in females, whereas the content of these PLs was higher at the 250 m depth compared to other sampled depths. The male and female contents, respectively, of minor PLs, such as lysophosphatidylcholine (LPC), phosphatidylinositol (PI), and phosphatidylserine (PS) were determined as 0.04–0.09 and 0.01–0.07, 0.01–0.06 and 0.003–0.05, and 0.01–0.02 and 0.002–0.02% dry weight, respectively. The PI and PS were closely correlated with the major PC and PEA (r within 0.91–0.98), while the correlations between these minor PLs and depth were r = −0.89 and −0.95, respectively. A significant dynamic of minor PL content in fish muscles with depth was only observed for PS in males. The dynamics of the LPC change was inversely proportional to the PC change.

The random forest algorithm applied to individual PL classes revealed two classifiers that defined the sample grouping and distribution—PEA and LPC.

### 3.4. Species-Specific Characteristics of the Fatty Acid Spectrum in Beaked Redfish, Its Sex Specificity, and Change Depth-Wise

**Figure 5:**
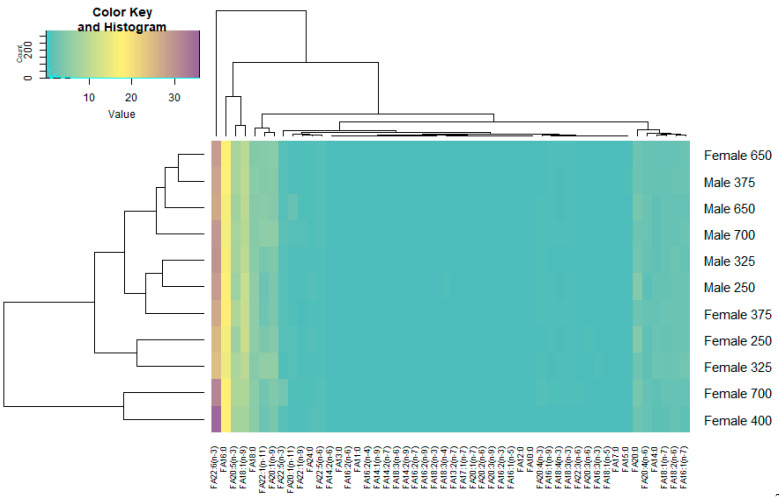
Heatmap of the qualitative and quantitative content of fatty acids (FAs) in the muscle tissue of male and female beaked redfish, *Sebastes mentella,* at different depths of the Irminger Sea (North Atlantic).

The FA profiling of the muscle of redfish males and females along the depth gradient was carried out, and the results are presented as a heat map (Figure 5). We found that the dominant fatty acids were PUFAs, among which n-3 PUFA prevailed due to docosahexaenoic acid (DHA—22:6(n-3)), that accounted for 27.23–29.83% and 24.45–35.78% of the total FA in the muscle tissues of males and females, respectively, and due to its metabolic precursor, eicosapentaenoic acid (EPA—22:5(n-3)), that contributed 6.87–8.07% and 6.75–8.91% of the total FA in males and females, respectively.

The content of DHA in fish muscle tissue increased (no significant changes were detected) depth-wise. The muscle n-6 PUFA content rose to 6% at some depths. The dominant n-6 PUFAs were arachidonic acid (ARA, 20:4(n-6)) and its metabolic precursor, linoleic acid (LIN, 18:2(n-6)), the contents of which varied within 1.4–2.39% and 1.73–2.14%, respectively, of the total FA in the muscles of males and within 2.03–2.73% and 1.87–2.55%, respectively, of the total FA in the muscles of females. No significant inverse correlation was demonstrated for the ARA and LIN contents in the muscles of both sexes with growing depths. The 20:4(n-6)/18:2(n-6) ratio (ARA to LIN ratio) reliably increased depth-wise.

Statistical analysis showed significant differences in the n-3/n-6 PUFA ratio, which reflected the competitive metabolic relationship between these FA families and, hence, the prevalence of one of the FA families, in the muscle tissue of males and females at the 250 and 700 m depths—7.32% for males and 20.55% for females versus 6.08% for males and 30.74% for females, respectively, of the total FA. Quantitatively, the second position belonged to saturated fatty acids (SFAs) due to palmitic (16:0), stearic (18:0), and eicosanoic (20:0) FAs, which amounted to 16.13–17.22% and 16.31–18.28%, 4.70–5.72% and 4.46–5.70%, and 2.64–4.35% and 2.73–4.46% of the total FA in males and females, respectively.

**Figure 6:**
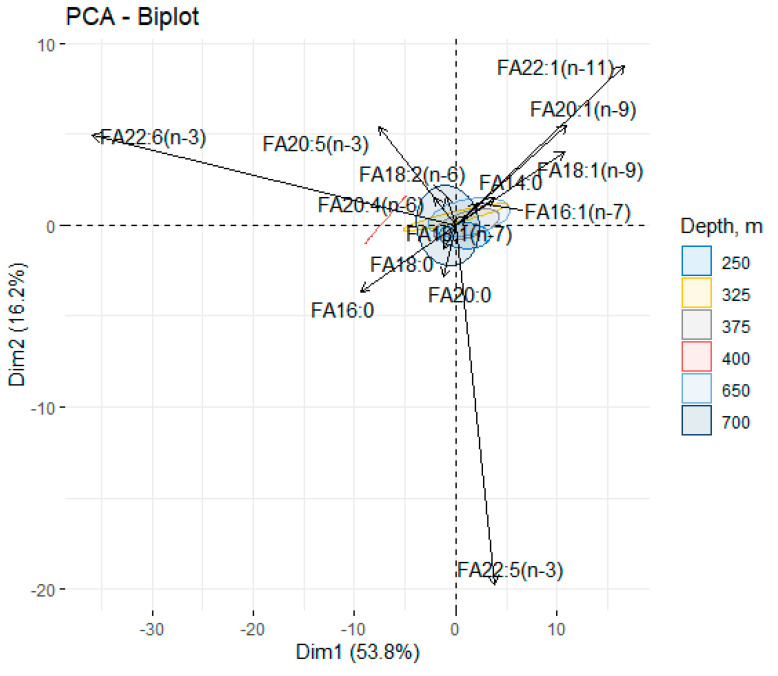
Principal component analysis of the fatty acid composition in the muscle tissue of male and female beaked redfish, *Sebastes mentella,* in the depth gradient of the Irminger Sea (North Atlantic).

The contents of monounsaturated fatty acids (MUFAs), primarily represented by oleic acid (18:1(n-9)), ranged within 9.62–11.25% and 7.75–11.81% of the total FA for males and females, respectively. In addition, the redfish muscle tissue contained zooplankton (copepod) 20:1(n-9) (3.8–5.63% and 3.53–5.19% of the total FA) and 22:1(n-11) (2.43–5.62% and 2.58–6.01% of the total FA) marker FAs in males and females, respectively. Multivariate statistical analysis based on principal components analysis (PCA) explained 70% of the total variance (Dim1—53.8; Dim2—16.8) and showed an absence of factor-induced differences (Figure 6). 

It was noted that as the depth decreased, the variance shifted along the 20:1(n-9) and 22:1(n-11) FA zooplankton (copepods) markers, whereas the shift with increasing depth was along DHA and ARA, which usually rose with depth. The contribution of the 16:0 (Dim.1 = -0.21) and 18:1(n-9) (Dim.1 = 0.24) FAs to the principal component indicated a decline in the oleic acid content and a rise in the palmitic acid content with depth. The ratio of the lipid metabolism rate (16:0/18:1(n-9)), however, only increased in females (from 1.41% to 2.13% of the total FA).

## 4. Discussion

Lipids are the principal structural and energetic components of marine organisms, the primary depots of which are muscle tissue and the liver [24,61]. Studies have revealed a relatively low content of TLs in the muscles of beaked redfish males and females (7.09–11.87 and 2.01–9.47% dry weight, respectively), which is regarded as a biotechnological characteristic of redfish. In terms of the fat/protein ratio, the North Atlantic *Sebastes* can be classified as a semi-fat fish (fat content: 3–6%), while fish such as the Greenland halibut (*Reinhardtius hippoglossoides*) and the Atlantic salmon (*Salmo salar*) are described as fat (fat contents >10% and >15%, respectively) [61,62].

The length–weight parameters of beaked redfish and their preferred depths are known to change with age; however, vertical circadian and seasonal (breeding) migrations continue [35,50]. In this study, we observed differently-directed depth-wise TL trends in the muscle of males and females, which pointed to sex-specific lipid accumulation patterns. The samples for this study were collected in June at the end of the larvae release season [35,63], when differences between sexes in TL levels are even greater. The absence of significant TL dynamics within either males or females across depths proves that TLs have a role in maintaining adequate metabolic functions in specific length–weight groups throughout the depth range and do not depend on the depth chosen by the fish.

One of the mechanisms for the biochemical adaptation of aquatic organisms, including fish, to deep-water life where food is at a deficit is to modify the set and ratio of neutral lipids in the body [64,65]. We found that Chol esters prevailed in redfish muscles over the storage TAGs, and the wax ester content was high, which is a characteristic trait of vertically migrating species. In addition to supplying an organism with energy, multifunctional Chol esters, as well as wax esters, participate in maintaining adequate buoyancy through involvement in compensatory adaptation mechanisms [14,15,66,67,68]. These mechanisms engage the alteration of the cell membrane fluidity and the implementation of the signaling and regulatory functions related to depth change, thus facilitating depth- (over hundreds of meters) and frequency-independent circadian migrations in the water column.

Arctic copepods in stress-induced conditions, such as starvation periods, have demonstrated the ability to hydrolyze wax esters to the more responsive molecules as TAGs [69]. The significant dominance of Chol esters found in males at the 250 m depth points to age-related changes in the deposition of this lipid class in muscles. The species-specificity of the storage lipids (wax esters or TAGs) is widely known for high-latitude zooplankton. Direct correlations were found between the depth at which these organisms inhabit, regardless of whether they perform vertical migrations, and the qualitative and quantitative composition of the lipids stored. When these organisms migrate to upper water layers, their lipid content declines (together with the wax ester content and NEFA content variation), supposedly in connection with the alteration of the rate of lipolysis relevant for maintaining the muscle contraction function and controlling the buoyancy type, which likely generates energy savings [70]. A similar mechanism was found to be enacted in the redfish male group at the 250 m depth. Younger redfish groups concentrate at smaller depths [35]. No such differences were found in females, possibly due to the greater diffusion of length–weight values over the depth gradient at the completion of the breeding season and start of feeding migration [49].

The contents of TAGs were found to increase with depth, most likely due to the accumulation of high-energy lipids and their FA components as strategic resources, as TAGs comprise a class of molecules with a high energy capacity (generating 2.5-times more energy than carbohydrates) that can be quickly mobilized from adipocytes [19]. The epipelagic zone and upper mesopelagic layers are where the bulk of zoo- and phyto-plankton, which synthesize high-energy FAs, are concentrated [64,65,71,72,73,74]. The depth range inhabited by beaked redfish in the Irminger Sea extends down to more than 1000 m [42,47,48], where a depth-wise food deficit may develop [64,65].

The key mechanisms for maintaining biomembrane integrity during adaptions to temperature and deep-water environments in fish include qualitative and quantitative modifications of PL composition, mainly through the dominant PC and PEA fractions (the most frequent mechanism in poikilotherms as measured by the PC/PEA and PEA/PC ratios), qualitative and quantitative modifications of minor PL, variations of the Chol and total PL contents (as indicated by changes in the Chol/PL molar ratio in membranes), and the qualitative modification of the FA components of PL—the so-called change in lipid unsaturation [2,5,9,75,76].

Minor PLs (PI and PS) were also found to participate in microviscosity regulation through the modification of the ion permeability and excitability of membranes, as well as the transmission of external signals into the cell [77]. This study detected an inverse correlation between PC, PEA, PS, and PI in redfish muscle tissue and the depth at which the fish lived, suggesting that these compounds are involved in the rearrangement of the membrane’s physicochemical state, securing the microenvironment homeostasis necessary for the functioning of membrane-bound enzymes and their ensembles.

However, another probable reason for the observed depth-related variations in the minor PI and PS, together with DAGs, is that these lipids take part in the regulation of protein kinase C activity in Ca^2+^ ion transport, which is particularly important for locomotion in environments with high hydrostatic pressure [78,79]. Importantly, PI moieties can be used in the organism as donors of DAGs and ARA for the synthesis of lipidic mediators in compensatory responses to external environmental impacts [80]. The variations detected in the minor LPC in the muscle tissue of the beaked redfish are directly linked to changes in the quantities of its metabolic precursor—PC.

LPC can be additionally synthesized through DAG hydrolysis [81]. A build-up of LPC in cells makes membranes more permeable to ions, and LPC break-up products (NEFAs, glycerophosphoric esters, and choline) can be used in the synthesis of biologically active compounds, namely hormones and neurotransmitters, that are needed for muscular activity and the coordination of body activities [82,83,84].

As demonstrated by statistical analysis, the strongest classifiers for both sexes of this species along the depth gradient were the metabolically interrelated DAGs, NEFAs, Chol esters, and wax esters. This indicates a correlation between these lipids and the depths at which the fish live and their role in maintaining the physiological and biochemical functions at different depths. Exceptional roles of DAGs and wax esters at the sampled depths were, however, found only for males.

Fatty acids, as integral components of lipids, are highly polyfunctional—their quantities and composition are directly dependent on the organism’s physiological status and the combination of environmental factors [9,85]. The high contents of essential omega-3 polyunsaturated DHA and EPA we observed in beaked redfish muscles comprises the species’ characteristic trait, and, as demonstrated previously for redfish [24] and other deep-water long-living fish species, the quantities of these acids increased with age.

The high contents of these acids and their variations under low temperatures and high pressures are some of the key mechanisms in the compensatory response to changes in the environment. Researchers have reported [1,86,87] that the content of the essential 22:6(n-3) FA in fish lipids is influenced by ambient temperature and depth (hydrostatic pressure), as well as by the natural mobility of fish. A previous study [88] revealed an elevation of the 22:6(n-3) level in the muscles of the arctoboreal daubed shanny, *Leptoclinus maculatus*, which lives at depths of 250–300 m.

More recently, researchers [11] found that lipid unsaturation in arctic fish was primarily maintained by means of DHA and EPA. This is related to the organism’s locomotor activity under certain conditions (pressure, temperature, currents, etc.) and is eventually targeted at the regulation of membrane microviscosity and, hence, the functional activity of membrane-bound enzymes in the context of overall metabolic activation [86]. This phenomenon is apparently based on a mechanism of engaging certain properties—an increased flexibility (associated with the membrane fluidity property) for the polyunsaturated 20:5(n-3)*cis*, 22:5(n-3)*cis*, and 22:6(n-3)*cis* chains compared to SFA.

When fish muscle tissue has to carry an increased metabolic load under specific environmental conditions (low temperatures, elevated dissolved oxygen concentration, salinity fluctuations, etc.), the relevant functioning of membranes is secured by controlling their thickness, fluidity, and resistance to mechanical impacts (during high protein activity), as well as by implementing the thermal insulation function for membrane-bound enzymes in certain lipid bilayer domains. Thus, the results obtained in this study, as well as earlier [11], regarding the FA status of deep-water fish in northern seas agree with the previously formulated concept of the functions of polyunsaturated chains [89,90,91]: the configuration of FAs in lipids defines the properties, functions, and biological effects of the respective lipids in the organism.

On the other hand, considering the elevated content of DHA and EPA, this species can be discussed as not only an important food resource but also a promising object for marine biotechnology. These PUFAs perform immunomodulation and general tonic effects on humans, and they are effectively used in the therapy of cardiovascular diseases and neurodegenerative disorders. A recent study [92] demonstrated the applicability and significance of marine sources of DHA, from which new high-activity compounds can be synthesized to promote in turn neuron regeneration after traumatic brain injury.

The opposite directions in which the contents of major omega-6 PUFAs (ARA and LIN) trend regarding the depth gradient may be due to the different food spectra of fish living at different depths. ARA content is known to be higher in demersal organisms [93,94]. The elevated content of 20:4(n-6) in males in our study was likely also connected with PI hydrolysis for the synthesis of various metabolites, including a variety of lipid mediators [85].

The high content of the palmitic 16:0 FA detected in our study pointed to a trophic link to krill (euphausiids)—a primary food item for the beaked redfish [53,95]. The depth-wise increase in the lipid metabolism index (16:0/18:1(n-9)) in females indicates an activation of the enzymatic complex for the de novo synthesis of one of the most universal MUFA—oleic acid (which is the main FA in storage lipids and is incorporated in biomembranes) in muscles [96,97,98].

MUFAs such as 20:1(n-9) and 22:1(n-11) are considered trophic biomarkers of zooplankton (copepods of the genus *Calanus*) [99,100]. Some studies [101,102] have demonstrated that the Σ22:1/Σ20:1 FA ratio is an informative parameter of the spatial distribution of copepods over the water column. The *de novo* synthesis of these FA takes place only in copepods. In our study, this ratio in the muscle tissue of beaked redfish from the sampled depths was >1 (only in males at the 650 m depth), <1 (in both females and males at the 250 and 375 m depths; only in females at the 400, 650, and 700 m depths; and only in males at the 325 m depth), and =1 (in females at the 325 m depth and in males at the 700 m depth), indicating variations in the contribution to the redfish diet of the copepod species known for these latitudes—*Calanus hyperboreus*, *Calanus glacialis*, and *Calanus finmarchicus*, respectively.

These results provide evidence for arguing about the dominant role of the widespread *C. glacialis* in the diet of males and, in particular, females across most of the sampled depths. The footprints of both the arctic deep-water *C. hyperboreus* (650 m) and the boreal *C. finmarchicus* (700 m) were seen only in males at substantial depths. The finding of the *C. finmarchicus* biomarker FA in the muscle tissue of redfish males was unusual and equivocal—whether these data reflect the ability of males to preserve these FAs within certain lipids and, thus, demonstrate a trophic link between the pelagic and the deep-water regions or they indicate the presence of C*. finmarchicus* at the 700 m depth remains to be clarified by further studies.

We currently lean more to the former explanation, mainly because the corresponding the Σ22:1/Σ20:1 FA ratio was found in *C. finmarchicus* females at the 325 m depth, which is a more realistic depth for this copepod species in the summer season. These speculations, however, need to be verified by further research. The lower biomarker footprint of *C. hyperboreus* and *C. finmarchicus* in redfish from greater depths, indicating a minor contribution of the copepods to the fish diet, has been previously reported by other authors [50,53,103]. Another perspective on the results is the selective accumulation of certain FAs in accordance with the compensatory function they perform—the utilization of zooplanktic and *de novo* synthesized MUFAs for energy-producing oxidation (within TAGs) or an increase in phytoplankton-derived PUFAs (DHA and EPA) for membrane state regulation.

## 5. Conclusions

A study of the lipid and fatty acids profile in the muscles of beaked redfish males and females from different depths in the Irminger Sea showed that there was no significant change of the total lipid content with depth. The total lipids dynamics with depth trended in the opposite directions by decreasing in males and increasing in females, indicating sex-specific differences for the lipid-level compensatory responses to vertical movements in the water column (vertical migration). This was corroborated by the prevalence of cholesterol esters over triacylglycerols and the high contents of wax esters.

One of the mechanisms for biochemical adaptation to deep-water environments with seasonal food deficiency in species performing vertical migrations is such species’ high levels of wax and cholesterol esters. In addition to providing for the organism’s energy demands, these lipid classes help maintain buoyancy, facilitating depth- (over hundreds of meters) and frequency-independent circadian migrations in the water column. The preference given to cholesterol esters as a source of energy, coupled with changes in structural phospholipid classes, arises from the polyfunctionality of this lipid—the utilization of certain components of the molecule in the energy metabolism (fatty acids) and for membrane viscosity regulation (cholesterol and phospholipid classes).

The inverse correlation detected between phospholipid classes in fish and the depth at which the fish lived suggests that they are involved in the rearrangement of the membrane physicochemical status, securing the environmental homeostasis necessary for the functioning of many membrane-bound enzymes and their ensembles. The changes identified in minor phospholipids (phosphatidylinositol and phosphatidylserine) could also be related to metabolic processes, as these lipid classes are donors for the synthesis of lipidic mediators.

The analysis of the FA spectrum of beaked redfish muscles revealed a high content of essential PUFAs, DHA (up to 30% of total FA) and EPA (up to 10% of total FA), and the contents of these FAs increased in fish inhabiting greater depths. The high contents of PUFAs, especially DHA and EPA, in muscles may be associated with an elevated or specific locomotor activity under certain conditions in a habitat (currents, turbulent flows, salinity, depth, and temperature).

Due to the high contents of DHA and EPA, redfish fillets have high nutritional and health value for humans, who are thus supplied with significant content of omega-3 PUFAs. DHA and EPA are known to produce immunomodulation and general tonic effects on humans, and they are effectively used in the therapy of cardiovascular diseases and cognitive disorders. The MUFAs in beaked redfish muscles include dietary markers of zooplankton (copepods)—20:1(n-9) and 22:1(n-11), the contents of in this tissue were found to be lower in fish sampled from greater depths, indicating a smaller contribution of zooplankton to fish diets in deep-water horizons.

Another perspective on the results is the selective accumulation of certain FAs in accord with the compensatory function they perform—the utilization of zooplanktic monounsaturated fatty acids to derive energy (within TAGs) or the participation of phytoplankton-derived PUFAs (DHA and EPA) in regulating the state of biomembranes. The contents of the latter compounds, as demonstrated for the beaked redfish in this paper, rise in fish living at greater depths.

## Figures and Tables

**Table 1:** Information on the studied individuals of beaked redfish (*Sebastidae*).

Sex	Quantity of Individuals	Body Length Min–Max, sm	Body Weight Min–Max, g	Date of Catch	№ Trawl	Latitude, N	Longitude, W	Depth, m
**Females**	1	39	696	June 17	12	64°6′	29°37′	375
	1	40	854	June 20	26	63°24′	37°34′	700
	1	38	614	June 21	29	63°5′	34°56′	325
	2	37–37	932–996	June 22	33	63°5′	31°54′	375
	2	40–40	890–940	June 23	36	63°5′	28°33′	700
	1	34	456	June 24	38	63°35′	27°18′	700
	2	38–38	666–710	June 25	41	62°31′	30°49′	400
	3	33–37	478–682	June 26	43	62°35′	32°28′	250
	1	32	440	June 27	48	62°35′	36°2′	250
	2	35–36	558–594	June 27	49	62°35′	37°7′	375
	4	28–33	302–432	June 28	52	62°36′	38°29′	700
	3	33–39	502–668	June 29	56	62°5′	36°35′	700
	1	36	546	July 03	69	61°45′	29°20′	650
	3	33–37	436–626	July 04	74	61°45′	33°13′	650
	1	38	696	July 12	94	61°26′	29°6′	650
**All**	28	28–40	302–996	June 17–July 12	12–94	61°26′–64°6′	27°18′–38°29′	250–700
**Males**	2	35–40	478–806	June 16	11	64°6′	31°22′	700
	1	36	604	June 17	12	64°6′	29°37′	375
	1	37	630	June 18	15	63°54′	27°57′	325
	2	32–35	352–476	June 19	22	63°35′	33°10′	700
	1	37	376	June 20	23	63°35′	35°5′	325
	5	35–37	528–664	June 21	29	63°5′	34°56′	325
	1	42	918	June 24	38	63°35′	27°18′	700
	1	36	708	June 26	43	62°35′	32°28′	250
	3	39–41	686–786	June 26	44	62°36′	33°24′	700
	9	28–34	282–550	June 27	48	62°35′	36°2′	250
	1	34	574	June 27	49	62°35′	37°7′	375
	5	32–36	354–556	June 28	52	62°36′	38°29′	700
	7	35–43	508–954	June 29	56	62°5′	36°35′	700
	1	35	538	July 03	69	61°45′	29°20′	650
	4	35–40	506–810	July 04	74	61°45′	33°13′	650
**All**	44	28–43	282–954	June 16–July 04	11–74	61°45′–64°6′	27°18′–38°29′	250–700
**Total**	72	28–43	282–996	June 16–July 12	11–94	61°45′–64°6′	27°18′–38°29′	250–700

## Data Availability

All data presented in the paper.

## References

[1] Kreps E.M. (1981). Lipids of Cellular Membranes: Evolution of Brain Lipids: Adaptive Function of Lipids.

[2] Sidorov V.S. (1983). Ecological Biochemistry of Fish.

[3] Henderson R.J. (1996). Fatty acid metabolism in freshwater fish with particular reference to polyunsaturated fatty acid. Arch. Anim. Nutr..

[4] Tocher D.R., Bell J.G., Dick J.R., Henderson R.J., McGhee F., Michell D., Morris P.C. (2000). Polyunsaturated fatty acid metabolism in Atlantic salmon (*Salmo salar*) undergoing parr-smolt transformation and the effects of dietary linseed and rapeseed oils. Fish Physiol. Biochem..

[5] Hochachka P.W., Somero G.N. (2002). Biochemical Adaptation: Mechanism and Process in Physiological Evolution.

[6] Arts M.T., Kohler C.C., Arts M.T., Brett M.T., Kainz M.J. (2009). Health and conditions in fish: The influence of lipids on membrane competency and immune response. Lipids in Aquatic Ecosystems.

[7] Murzina S.A., Nefedova Z.A., Nemova N.N. (2012). Effects of fatty acids (biomarkers of food sources) on the mechanisms of fish adaptation in high latitudes. Trans. KarRC RAS.

[8] Pavlov D.S., Nemova N.N., Kirillova E.A., Kirillov P.I., Nefedova Z.A., Murzina S.A. (2012). Lipid Content in the Young of the Year Sockeye Salmon *Oncorhynchus nerka* during Feeding Migration (the Ozernaya River, Western Kamchatka). Dokl. Biol. Sci..

[9] Nemova N.N., Nefyodova Z.A., Murzina S.A. (2014). Lipid patterns early in Atlantic salmon, *Salmo salar* L., ontogeny. Trans. KarRC RAS.

[10] Berge J., Renaud P.E., Darnis G., Cottier F., Last K.S., Gabrielsen T.M., Johnsen G., Seuthe L., Wes1awski J.M., Leu E. (2015). In the dark: A review of ecosystem processes during the Arctic polar night. Prog. Oceanogr..

[11] Murzina S.A., Pekkoeva S.N., Kondakova E.A., Nefedova Z.A., Filippova K.A., Nemova N.N., Orlov A.M., Berge J., Falk-Petersen S. (2020). Tiny but Fatty: Lipids and Fatty Acids in the Daubed Shanny (*Leptoclinus maculatus*), a Small Fish in Svalbard Waters. Biomolecules.

[12] Somero G.N. (1992). Adaptation to high hydrostatic pressure. Annu. Rev. Physiol..

[13] Gerringer M.E., Drazen J.C., Yancey P.H. (2017). Metabolic enzyme activities of abyssal and hadal fishes: Pressure effects and a re-evaluation of depth-related changes. Deep-Sea Res. Part 1.

[14] Neighbors M.A. (1988). Triacylglycerols and wax esters in the lipids of deep midwater teleost fishes of the Southern California Bright. Mar. Biol..

[15] Phleger C.F., Nelson M.M., Mooney B.D., Nichols P.D. (1999). Wax esters versus triacylglycerols in myctophid fishes from the Southern Ocean. Antarct. Sci..

[16] Tocher D.R. (2003). Metabolism and functions of lipids and fatty acids in teleost fish. Rev. Fish. Sci..

[17] Shulman G.E., Jakovleva K.K. (1983). Hexaenoic Acid and Natural Mobility of Fish. Biol. Bull. Rev..

[18] Zabelinscii S.A., Chebotareva M.A., Brovcina N.B., Krivchenko A.I. (1995). On “adaptive signaling” of the composition of conformational states of fatty acids in membrane lipids of fish gills. J. Evol. Biochem. Physiol..

[19] Lapin V.I., Shatunovskii M.I. (1981). Features of the composition, physiological and ecological significance of fish lipids. Biol. Bull. Rev..

[20] Velansky P.V., Kostetsky E.Y. (2008). Lipids of marine cold-water fishes. Russ. J. Mar. Biol..

[21] Joensen H., Grahl-Nielsen O. (2000). Discrimination of *Sebastes viviparus*, *Sebastes marinus* and *Sebastes mentella* from Faroe Islands by chemometry of the fatty acid profile in heart and gill tissues and in the skull oil. Comp. Biochem. Physiol. Part B.

[22] Joensen H., Grahl-Nielsen O. (2001). The redfish species *Sebastes viviparus*, *Sebastes marinus* and *Sebastes mentella* have different composition of their tissue fatty acids. Comp. Biochem. Physiol. Part B.

[23] Joensen H., Grahl-Nielsen O. (2004). Stock structure of *Sebastes mentella* in the North Atlantic revealed by chemometry of the fatty acid profile in heart tissue. ICES J. Mar. Sci..

[24] Petursdottir H., Gislason A., Falk-Petersen S. (2008). Lipid classes and fatty acid composition of muscle, liver and skull oil in deep-sea redfish *Sebastes mentella* over the Reykjanes Ridge. J. Fish Biol..

[25] Nefedova Z.A., Murzina S.A., Veselov A.E., Ripatti P.O., Nemova N.N. (2014). Heterogeneity of Lipids and Fatty Acids of Fingerlings of the Atlantic Salmon *Salmo Salar* L. Different in Weight and Size. Contemp. Probl. Ecol..

[26] Nefedova Z.A., Murzina S.A., Pekkoeva S.N., Nemova N.N. (2018). Comparative Characteristic of Fatty Acids Profiles of Smolts of Brown Trout *Salmo salar* L. and Atlantic Salmon *Salmo salar* During Smoltification (Indera River, White Sea Basin). Biol. Bull.

[27] Murzina S.A., Nefedova Z.A., Ripatti P.O., Nemova N.N., Pekkoeva S.N. (2012). Lipid and fatty acid content of the White Sea herring (*Clupea pallasi marisalbi* Berg) in relation to geographical distribution and environment in the White Sea (Northern Karelia, Russia). Comp. Biochem. Physiol. Part A Mol. Integr. Physiol..

[28] Murzina S.A., Nefedova Z.A., Pekkoeva S.N., Ruokolainen T.R., Ripatti P.O., Nemova N.N., Semushin A.V. (2016). Lipids and fatty acids of the White Sea herring *Clupea pallasi marisalbi* Berg (Clupeiformes, Clupeidae) from different habitats of the White Sea. Fishes.

[29] Nemova N.N., Murzina S.A., Nefedova Z.A., Pekkoeva S.N., Ropatti P.O. (2015). Lipid status of larvae and adults of the White Sea herring *Clupea pallasii marisalbi* Berg (Clupeiformes, Clupeidae). Dokl. Biochem. Biophys..

[30] Johnson L., Zhu J., Scott E.R., Wineinger N.E. (2015). An Examination of the Relationship between Lipid Levels and Associated Genetic Markers across Racial/Ethnic Populations in the Multi-Ethnic Study of Atherosclerosis. PLoS ONE.

[31] Zubair N., Graff M., Luis Ambite J., Bush W.S., Kichaev G., Lu Y., Manichaikul A., Sheu W.H., Absher D., Assimes T.L. (2016). Fine-mapping of lipid regions in global populations discovers ethnic-specific signals and refines previosly identified lipid loci. Hum. Mol. Genet..

[32] Sidore C., Busonero F., Maschio A., Porcu E., Naitza S., Zoledziewska M., Mulas A., Pistis G., Steri M., Danjou F. (2015). Genome sequencing elucidates Sardinian genetic architecture and augments association analyses for lipid and blood inflammatory markers. Nat. Genet..

[33] Reis-Santos P., Tanner S.E., Aboim M.A., Vasconcelos R.P., Laroche J., Charrier G., Perez M., Presa P., Gillanders B.M., Cabral H.N. (2018). Reconciling differences in natural tags to infer demographic and genetic connectivity in marine fish populations. Sci. Rep..

[34] Xu S., Song N., Zhao L., Cai S., Han Z., Gao T. (2017). Genomic evidence for local adaptation in the ovoviviparous marine fish *Sebasticus marmoratus* with a background of population homogeneity. Sci. Rep..

[35] Saborido-Rey F., Garabana D., Stransky C., Melnikov S., Shibanov V. (2004). Review of the population structure and ecology of *S. mentella* in the Irminger sea and adjacent waters. Rev. Fish Biol. Fish..

[36] Pedchenco A.P. (2005). The role of interannual environmental variations in the geographic range of spawning and feeding concentrations of redfish *Sebastes mentella* in the Irminger Sea. ICES J. Mar. Sci..

[37] Sigurŏsson T., Kristinsson K., Ratz H.J., Nedreaas K.H., Melnikov S.P., Reinert J. (2006). The fishery for pelagic redfish (*Sebastes mentella*) in the Irminger Sea and adjacent waters. ICES J. Mar. Sci..

[38] Planque B., Kristinsson K., Astakhov A., Bernreuther M., Bethke E., Drevetnyak K., Nedreaas K., Reinert J., Rolskiy A., Sigurdsson T. (2013). Monitoring beaked redfish (*Sebastes mentella*) in the North Atlantic, current challenges and future prospects. Aquat. Living Resour..

[39] Krovnin A.S., Melnikov S.P., Kivva K.K., Artemenkov D.V., Moury G.P. (2017). Influence of variability of oceanological conditions on redfish in the North Atlantic pelagial. Trudy VNIRO.

[40] Travin V.I. (1951). New species of the sea bass in Barents sea (*Sebastes mentella* Travin sp. nov.). Dokl. Akad. Nauk. SSSR.

[41] Templeman W. (1959). Redfish distribution in the North Atlantic. Bull. Fish. Res. Board Can..

[42] Hureau J.-C., Litvinenko N.I., Whitehead P.J.P., Bauchot M.-L., Hureau J.-C., Nielsen J., Tortonese E. (1986). Scorpaenidae. Fishes of the North-Eastern Atlantic and the Mediterranean (FNAM).

[43] Barsukov V.V. (2003). Annotated and Illustrated Check-List of Rockfishes of the World.

[44] Zakharov G.P. (1964). Redfish Above the Ocean Depths. ICNAF Res. Bull..

[45] Pavlov A., Mamylov V., Noskov A. (1989). Results of the USSR Investigations of Sebastes Mentella Travin in 1981–1988 (ICES Subareas XII, XIV).

[46] Magnússon J., Magnússon J.V. (1995). Oceanic redfish (*Sebastes mentella*) in the Irminger Sea and adjacent waters. Scentia Mar..

[47] Barsukov V.V., Litvinenko N.I., Serebryakov V.P. (1984). Manual for the identification of redfish species of the North Atlantic and adjacent areas. AtlantNIRO. Can. Transl. Fish. Aquat. Sci..

[48] Pavlov A.I. (1988). Distribution and Behavior of Redfish (Sebastes mentella Travin) on the Reykjanes Ridge as Observed from the Underwater Vehicle “Sever-2”.

[49] Melnikov S.P. (2006). North Atlantic Ocean Deep-Sea Redfish: Biology and Fisheries.

[50] Bakay Y.I., Melnikov S.P. (2008). Biological and ecological characteristics of deepwater redfish *Sebastes mentella* (Scorpaenidae) at different depths in the pelagial of the Irminger Sea. J. Ichthyol..

[51] Stefansson M.O., Sigurdsson T., Pampoulie C., Danielsdottir A.K., Thorgilsson B., Ragnarsdottir A., Gislason D., Coughlan J., Cross T.F., Bernatchez L. (2009). Pleistocene genetic legacy suggests incipient species of *Sebastes mentella* in the Irminger Sea. Heredity.

[52] Filina E.A., Rolskiy A.Y., Bakay Y.I., Popov V.I., Makeenko G.A. (2017). Features of the reproductive cycle in females of the beaked redfish *Sebastes mentella* (Sebastidae). J. Ichthyol..

[53] Petursdottir H., Gislason A., Falk-Petersen S., Hop H., Svavarsson J. (2008). Trophic interaction of the pelagic ecosystem over the Reykjanes Ridge as evaluated by fatty acid and stable isotope analyses. Deep-Sea Res. Part II.

[54] Barsukov V.V., Litvinenko N.I., Serebryakov V.P. (1984). Methods of Determination of Redfish Species of the Northern Part of the Atlantic Ocean and Adjacent Sees.

[55] (2015). Manual for the International Deep Pelagic Ecosystem Survey in the Irminger Sea and Adjacent Waters.

[56] Folch J., Lees M., Sloan-Syanley G.H. (1957). A simple method for the isolation and purification of total lipids from animal tissue (for brain, liver and muscle). J. Biol. Chem..

[57] Olsen R.E., Henderson R.J. (1989). The rapid analysis of neutral and polar marine lipids using double development HPTLC and scanning densitometry. J. Exp. Mar. Biol. Ecol..

[58] Hellwig J. (2005). Defining Parameters for A Reproducible TLC-Separation of Phospholipids Using ADC 2. Ph.D. Thesis.

[59] Kabakoff R., Volkova P.A. (2014). R in Action: Data Analysis and Graphics with R.

[60] Bruce A., Bruce P., Bruce P., Bruce A. (2018). Practical Statistic for Data Scientists.

[61] Karl H., Numata J., Lahrssen-Wiederholt M. (2018). Variability of fat, water and protein content in the flesh of beaked redfish (*Sebastes mentella*) and Greenland halibut (*Reinhardtius hippoglossoides*) from arctic fishing grounds. J. Consum. Prot. Food Saf..

[62] Nemova N.N. (2016). Ecological and Biochemical Status of Juvenile Atlantic Salmon Salmo Salar L. from Some Rivers of the White Sea Basin.

[63] Melnikov S.P. (2016). Intraspecies structure of beaked redfish *Sebastes mentella* of the Atlantic and Arctic oceans. J. Ichthyol..

[64] Lee R.F., Nevenzel J.C., Paffenhöfer G.-A. (1971). Importance of wax esters and other lipids in the marine food chain: Phytoplankton and copepods. Mar Biol..

[65] Wang F., Wu Y., Chen Z., Zhang G., Zhang J., Zheng S., Katter G. (2019). Trophic interactions of mesopelagic fishes in the South China Sea illustrated by stable isotopes and fatty acids. Front. Mar. Sci..

[66] Russ T.S., Linberg G.U. (1971). Modern understanding of the natural system of living fish. J. Ichthyol..

[67] Perevozchikov A.P. (2008). Sterols and their transport in animal development. Russ. J. Dev. Biol..

[68] Ozdemir N.S., Parrish C.C., Parzanini C., Mercier A. (2019). Neutral and polar lipid fatty acids in five families of demersal and pelagic fish from the deep Northwest Atlantic. ICES J. Mar. Sci..

[69] Sargent J.R. (1978). Marine wax esters. Sci. Progr..

[70] Shchepkina A.M., Trusevich V.V., Pavlovskaya T.Y. (1991). Peculiarities of lipid composition in some representatives of the mass species of tropical zooplancton from the Atlantic and Indian Ocean. Ecol. Sea.

[71] Dalsgaard J., St. John M., Kattner G., Muller-Navarra D., Hagen W. (2003). Fatty acid trophic markers in the pelagic marine environment. Adv. Mar. Biol..

[72] Gislason A. (2003). Life-cycle strategies and seasonal migrations of oceanic copepods in the Irminger Sea. Hydrobiologia.

[73] Anderson T.R., Martin A.P., Lampitt R.S., Trueman C.N., Henson S.A., Mayor D. (2019). Quantifying carbon fluxes from primary production to mesopelagic fish using a simple food web model. ICES J. Mar. Sci..

[74] Berge J., Geoffroy M., Daase M., Cottier F., Priou P., Cohen J.H., Johnsen G., McKee D., Kostakis I., Renaud P.E. (2020). Artificial light during the polar night disrupts Arctic fish and zooplankton behaviour down to 200 m depth. Commun. Biol..

[75] Gennis R. (1997). Biomembranes: Molecular Structure and Function.

[76] Boldyrev A.A., Kyayvaryainen E.I., Ilyukha V.A. (2006). Biomembranology: A Textbook.

[77] Makarova I.I., Golovko M.Y. (2001). Asymmetry of the source of secondary messengers—Phosphatidylinositol of the cerebral cortex of rats with an increase in geomagnetic activity. Actual Problems of Functional Interhemispheric Asymmetry: Conference 13–14 December 2001, Moskow.

[78] Colman J., Rem K.-G. (2009). Visual Biochemistry.

[79] Sandel E., Nixon O., Lutzky S., Ginsbourg B., Tandler A., Uni Z., Koven W. (2010). The effect of dietary phosphatidylcholine/phosphatidylinositol ratio on malformation in larvae and juvenile gilthead sea bream (*Sparus aurata*). Aquaculture.

[80] Kim Y.J., Guzman-Hernandez M.L., Balla T. (2013). Inositol lipid regulation of lipid transfer in specialized membrane domains. Trends Cell Biol..

[81] Aarsman A.J., van den Bosch H. (1980). Does de novo synthesis of lysophosphatidylcholine occur in rat lung microsome?. Biochim. Biophys. Acta.

[82] Dobrynina V.I. (1976). Biological Chemistry.

[83] Osadchaya L.M., Galkina O.V., Eshchenko N.D. (2004). Effect of Corazole on the Activity of Na+ -K+ ATP—The Basics and the Intensity of Lipid Peroxidation in Neurons and Neuroglia: Biochemical and Molecular-Biological Foundations of Physiological Functions.

[84] Berdichevets I.N., Tyazhelova T.V., Shimshilashvili K.R., Rogaev E.I. (2010). Lysophosphatidic acid is a lipid mediator with wide range of biological activities. Biosynthetic pathways and mechanism of action. Biochemistry.

[85] Tocher D.R., Fonseca-Madrigal J., Bell J.G., Dick J.R., Henderson R.J., Sargent J.R. (2002). Effect of diets containing linseed oil on fatty acid desaturation and oxidation in hepatocytes and intestinal enterocytes in Atlantic salmon (*Salmo salar*). Fish Physiol. Biochem..

[86] Shulman G.E., Yuneva T.V. (1990). Role of docosahexaenoic acid in adaptations fishes (review). Hydrobiol. J..

[87] Kanazawa A. (1997). Effects of docosahexaenoic acid and phospholipids on stress tolerance of fish. Aquaculture.

[88] Murzina S.A. (2010). The Role of Lipids and Their Fatty Acid Components in Biochemical Adaptations of the Spotted Lumpen *Leptoclinus Maculatus* F. Spitsbergen. Ph.D. Thesis.

[89] Rabinovich A.L., Ripatti P.O. (1994). Polyunsaturated carbon chins of lipids: Structure, properties, functions. Biol. Bull. Rev..

[90] Rabinovich A.L., Ripatti P.O., Balabaev N.K. (2004). Molecular parameters of hydrated bilayers of unsaturated phosphatidylcholines. Russ. J. Phys. Chem. A.

[91] Rabinovich A.L., Ivanov V.A., Rabinovich A.L., Hohlov A.R. (2009). Chain molecules as components of membrane systems: Computer modeling. Computer Simulation Methods for Researching Polymers and Biopolymers.

[92] Ponomarenco A.I., Tyrtyshnaia A.A., Pislyagin E.A., Dyuizen I.V., Sultanov R.M., Manzhulo I.V. (2021). N-docosahexaenoylethanolamine reduces neuroinflammation and cognitive impairment after mild traumatic brain injury in rats. Sci. Rep..

[93] Suhr S.B., Pond D.W., Gooday A.J., Smith C.R. (2003). Selective feeding by benthic foraminifera on phytodetritus on the western Antarctic Peninsula shelf: Evidence from fatty acid biomarker analysis. Mar. Ecol. Prog. Ser..

[94] Hudson I.R., Pond D.W., Billett D.S.M., Tyler P.A., Lampitt R.S., Wolff G.A. (2004). Temporal variations in fatty acid composition of deep-sea holothurians: Evidence of bentho-pelagic coupling. Mar. Ecol. Prog. Ser..

[95] Gershanovich A.D. Lipid mobilization during early development of turgeons. Proceedings of the First International Symposium Sturgeon.

[96] Sargent J.R., McEvoy L.A., Estevez A., Bell G., Bell M., Henderson J., Tocher D. (1987). Requirements, presentation and sources of polyunsaturated fatty acids in marine fish larval feeds. Aquaculture.

[97] Falk-Petersen S., Sargent J.R., Henderson J., Hagseth E.N., Hop H., Okolodkov Y.B. (1998). Lipids and fatty acids in ice algae and phytoplankton from the Marginal Ice Zone in the Barents Sea. Polar. Biol..

[98] Nelson M.M., Mooney B., Nichols J.D., Phleger C.F. (2001). Lipids of Antarctic Ocean amphipods: Food chain interactions and the occurrence of novel biomarkers. Mar. Chem..

[99] Lee R.F. (1974). Lipid composition of the copepod *Calanus hyperboreas* from the Arctic Ocean. Changes with depth and season. Mar. Biol..

[100] Hagen W., Auel H. (2001). Seasonal adaptations and the role of lipids in oceanic zooplankton. Zoology.

[101] Sargent J.R., Falk-Petersen S. (1988). The lipid biochemistry of calanoid copepods. Hydrobiologia.

[102] Scott C.L., Kwasniewski S., Falk-Petersen S., Sargent J.R. (2002). Species differences, origins and functions of fatty alcohols and fatty acids in the wax esters and phospholipids of *Calanus hyperboreus*, *C. glacialis* and *C. finmarchicus* from Arctic waters. Mar. Ecol. Prog. Ser..

[103] Dolgov V.A., Rolsky A. Yu., Popov V.I. Feeding of redfish *Sebastes mentella* in the Irminger Sea—What do the data on feeding show?. Proceedings of the ICES Annual Science Conference.

